# Undifferentiated embryonal sarcoma of the liver with epithelioid features in an adult patient: A case report

**DOI:** 10.1097/MD.0000000000028265

**Published:** 2021-12-17

**Authors:** Peng Jiang, Yan Jiao, Chun-Yuan Niu, Ya-Hui Liu

**Affiliations:** Department of Hepatobiliary and Pancreatic Surgery, First Hospital of Jilin University, Changchun, Jilin, China.

**Keywords:** case report, epithelioid differentiation, liver cancer, undifferentiated embryonal sarcoma of the liver, undifferentiated sarcoma

## Abstract

**Introduction:**

Undifferentiated embryonal sarcoma of the liver (UESL) is a rare form of liver malignancy, with most cases reported in the pediatric population. This disease is extremely uncommon in adults. Herein, we report the first case of UESL with epithelioid features in an adult patient.

**Patient concerns:**

A 50-year-old man was admitted to our hospital due to epigastric pain.

**Diagnosis and interventions:**

Computed tomography and magnetic resonance imaging revealed a space-occupying lesion in the right lobe of the liver. A right hemihepatectomy was performed. Postoperative pathological and immunohistochemical examinations confirmed the diagnosis of UESL and features of epithelioid differentiation.

**Outcomes:**

The patient recovered well and refused adjuvant therapy. Unfortunately, the patient died of tumor recurrence 3 months after hospital discharge.

**Conclusion:**

UESL is a rare form of liver cancer, with most cases reported in the pediatric population. This case study highlights an extremely uncommon case of UESL with epithelioid features and a very poor prognosis. The findings suggest that complete intraoperative resection and postoperative adjuvant therapy should be considered to improve the prognosis of adult patients with UESL with epithelioid features.

## 1 Introduction

Undifferentiated embryonal sarcoma of the liver (UESL) is a rare hepatic mesenchymal tumor with a high potential for invasion and poor prognosis.^[1]^ Most UESL cases have been reported in the pediatric population at the ages of 6 to 10, and the disease is extremely uncommon in adults (2). Since UESL was first described in 1978,^[2]^ approximately 89 adult UESL cases have been reported across the world.^[3]^ To date, the pathogenesis of UESL remains obscure, and both diagnosis and treatment remain clinically challenging. Previous studies have shown that the 5-year survival rate in pediatric patients is approximately 86%.^[4,5]^ The diagnosis of UESL often relies on pathological and immunohistochemical examinations, laboratory tests, and preoperative imaging findings. Early detection, complete surgical resection, and adjuvant therapy are key to achieving good clinical outcomes.^[4,6]^

In this case study, we present an extremely rare case of UESL with epithelioid features in a 50-year-old man. The findings in this case report may add to the understanding of adult UESL and improve the treatment strategy for the disease.

## 2 Case presentation

A 50-year-old man was admitted to our hospital with epigastric pain that lasted for more than 2 days. The patient had a history of hepatitis B-associated liver cirrhosis for more than 20 years and hyperlipidemia for more than 7 years. Neither condition was treated.

Physical examination revealed mild epigastric tenderness. Laboratory tests showed the following: γ-glutamyl transpeptidase of 120.2 units/L (U/L), negative for alpha-fetoprotein, abnormal prothrombin (67.65 ng/mL, and Golgi protein-73 (252.50 ng/mL. The liver reserve function test showed that the retention rate of indocyanine green at 15 minute was 5.1%. Child-Pugh liver function was classified as grade A. Inflammatory factors and tumor markers were within the normal ranges.

**Figure 1:**
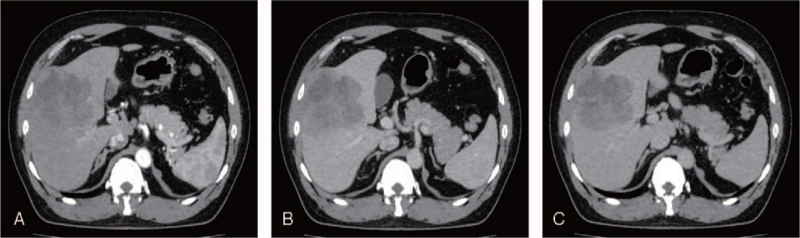
Three-phase enhanced CT of the abdomen. (A) Arterial phase CT image showed irregular patch-like enhancement; CT images in (B) the portal venous phase, and (C) the delayed phase.

**Figure 2:**
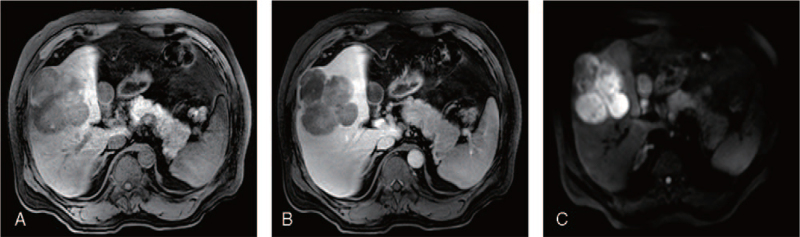
Magnetic resonance imaging (MRI) of the liver, gallbladder, and spleen. (A) T1 weighted (T1W1) image; (B) T2 weighted (T2W1) image; (C) diffusion weighted imaging (DWI).

Three-phase enhanced computed tomography (CT) (Fig. 1) of the abdomen revealed a mass-like low-density shadow in the right lobe of the liver protruding from the liver surface. The size was approximately 9.2 × 9.6 cm, and the internal density was uneven. CT scans showed fusion of multiple round-like low-density foci and irregular patchy enhancement in the arterial phase. Plain scan, enhanced (gadoxetic acid disodium injection, Pumeixian), and diffusion-weighted magnetic resonance imaging (Fig. 2) of the liver, gallbladder, and spleen showed multiple nodules and masses with abnormally high signals in the right lobe of the liver, some of which protruded beyond the liver surface with a size of approximately 0.9 to 9.1 cm. No significant lesions were observed on thoracic CT. Primary liver cancer was initially considered, and bleeding was observed in larger lesions.

Right hemihepatectomy was performed with the consent of the patient and his family members for medical treatment. During the laparoscopic surgery, a large mass with a volume of 10 cm × 7.5 cm × 7.5 cm infiltrating the adjacent tissue was found surrounded by a local fibrous capsule. Invasion into the hepatic capsule, tumor-infiltrating blood vessels, no obvious tumor infiltration into the nerves, and multiple tumor nodules (0.5-2.5 cm in diameter) in omental adipose tissue were observed. The final surgical margin was negative.

**Figure 3:**
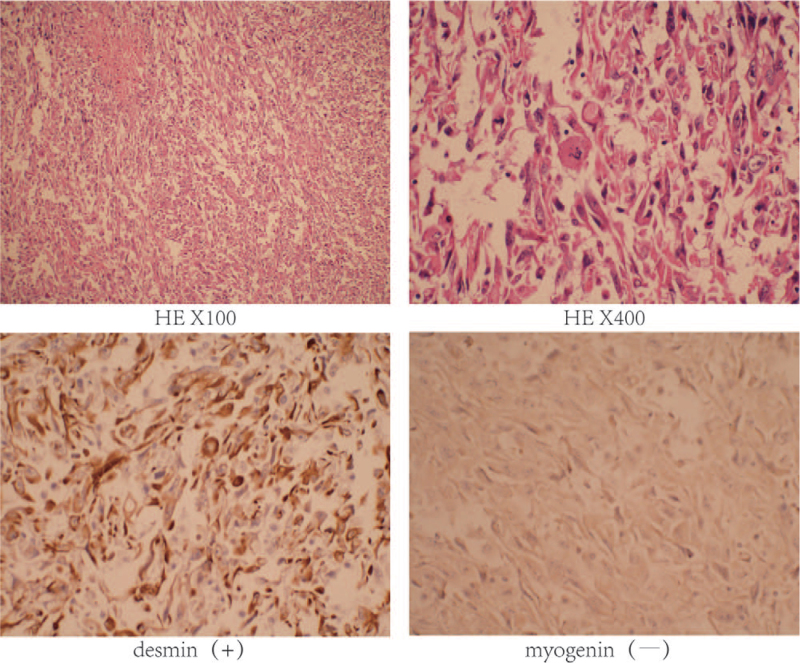
Histological and immunohistochemical examinations. Postoperative histological and immunohistochemical examinations were performed and the findings supported the diagnosis of UESL with epithelioid features. Immunohistochemistry: desmin (+), myogenin (–).

Postoperative pathological and immunohistochemical examinations were performed. The results indicated that the lesion was negative for CD31, CD34, SMA, CD117, CK7, Dog-1, GPC-3, S100, calretinin, CD21, WT-1, and myogenin, and positive for desmin (+), FLI-1 (±), Ki-67 (30% +), vimentin (+), CK-pan (+), and CK5/6 (±). Based on the postoperative pathological and immunohistochemical examinations (Fig. 3), a final diagnosis of UESL with epithelioid features was made.

The patient recovered well with favorable liver function after the operation, but refused adjuvant therapy (chemotherapy or targeted therapy) for financial reasons. Unfortunately, the patient died of tumor recurrence 3 months after hospital discharge due to extensive intra-abdominal tumor recurrence.

## 3 Discussion

UESL has been described as a rare mesenchymal malignant tumor that mostly occurs in children aged 6 to 10 years and is rarely observed in adults.^[1]^ In this case study, we encountered an extremely uncommon case of UESL with specific features of epithelioid differentiation in a 50-year-old male patient. To the best of our knowledge, this is the first reported case of adult UESL with epithelioid features.

The diagnosis of UESL, especially in the early stage, remains a clinical challenge, mainly because UESL can be asymptomatic or cause nonspecific symptoms (e.g., nausea, vomiting, weight loss, fever, abdominal pain, and distension). In addition, most laboratory indicators are negative, such as liver function and tumor markers (e.g., alpha-fetoprotein and carcinoembryonic antigen). In some patients, CA125 and CA19-9 levels were slightly elevated. Biopsy is not suitable for cystic solid tumor.^[7]^ Indeed, the success rate of preoperative diagnosis is low. The diagnosis of UESL mainly relies on postoperative pathology and immunohistochemistry results from surgically resected lesions. At present, early diagnosis, complete surgical resection, and postoperative adjuvant therapy are key to achieving superior clinical outcomes.^[8]^ It has been noted that the right lobe of the liver is a common site for UESL.^[9]^ In the current case, the symptoms included loss of appetite and mild abdominal pain. Laboratory tests showed an elevated level of γ-glutamyl transpeptidase, which could be mainly attributed to the rapid growth of the large tumor and invasion of the liver surface visceral peritoneum, and that the cause of fatty liver was more serious. Additionally, UESL has no characteristic imaging findings.^[1]^ For instance, on CT, UESL often appears as an irregular mass-like low-density shadow, uneven internal density, solid and cystic, and more robust components can be seen on ultrasound. On magnetic resonance imaging, UESL has low or slightly high signal intensity on T1WI and abnormal signal intensity on T2W1. These nonspecific imaging features often lead to a misdiagnosis of UESL as a liver abscess. In pediatric patients, UESL is often preoperatively misdiagnosed as metastatic liver cancer, hepatoblastoma, or biliary tree embryonal rhabdomyosarcoma. Notably, the main differential diagnosis in adults is hepatocellular carcinoma, specifically the gastrointestinal scrotal tumor, angiolipoma, leiomyosarcoma, liposarcoma, angiosarcoma, epithelioid hemangioendothelioma, and malignant melanoma variants.^[6]^ This study considers that we should be alert to UESL when the following characteristics exist: The tumor is large, well-bounded, and filled with cysts with uneven density, showing room; the density of the cystic part of the tumor was low, and there was a slightly high-density flocculent or cable separation, or could have mural nodules; cystic partial enhancement was unknown after enhanced scanning. There was mild and moderate uneven enhancement of the parenchyma and septa. In addition, the myxoid matrix of the inner cystic part of UESL is rich in hydrophilic acid mucopolysaccharides, which continuously absorb water, resulting in fluid appearance on CT, and in ultrasound, exhibiting an echo zone.^[10]^ It has been shown that high-grade undifferentiated cells in UESL are histologically heterogeneous and vary according to the examination area. As such, some areas may contain relatively uniform undifferentiated cells, while other areas may contain extensive pleomorphic cells. Considerable necrotic areas can be found in most specimens, and mitosis is common.^[11]^

It may merit attention that the findings suggested the present case of UESL had a high degree of malignancy and particularly strong infiltration. Multiple vascular infiltrations were found in the specimen, and there was a local fibrous capsule-infiltrating growth invading the liver capsule, which often predicts a poor prognosis. Currently, there is no distinctive immunohistochemical method for identifying UESL, and most of the immunohistochemical tests only rule out other diagnoses. Actin, vimentin, CD68, BCL-2, desmin, CD10, and positive variable expression of α-1-antitrypsin and α-1-antichymotrypsin in eosinophilic glomeruli are common in UESL.^[12]^ A few studies have also shown positive reactions to Glypican 3 and CD117.^[13]^ However, S100, MART1, myoglobin, ALK-1, β-catering, and HepPar1 are usually negative.

Previous studies in pediatric patients with UESL demonstrated that the 5-year survival rate was approximately 86%.^[4,5]^ Due to the limited number of adult UESL cases worldwide, the exact 5-year survival rate in adult patients with UESL remains unclear. At present, complete surgical resection in combination with postoperative chemotherapy is recognized as a treatment scheme to improve the clinical outcomes of UESL patients,^[14]^ but the chemotherapy regimen has not yet been unified. In addition, patients with UESL and tumor metastasis receive radiotherapy. However, there is no definitive treatment for UESL, and more case studies are needed to reach a consensus. For unresectable UESL, a small proportion of patients underwent orthotopic liver transplantation. According to the existing data, the median survival is 28.5 months, and adjuvant chemotherapy after liver transplantation is still recommended.^[4,15–17]^ Orthotopic liver transplantation is considered an alternative treatment for patients with indications for radical resection because of organ scarcity. In this case, we adopted surgical resection for the treatment of UESL without subsequent adjuvant chemotherapy for financial reasons. Unfortunately, the patient died of tumor recurrence 3 months after hospital discharge due to extensive intra-abdominal tumor recurrence, which is much shorter than the average survival for existing patients.^[18,19]^

## 4 Conclusion

Taken together, UESL with epithelioid features caused mild symptoms with nonspecific imaging findings in the present case. Therefore, preoperative diagnosis is challenging, and the diagnosis can be confirmed only by postoperative pathology and immunohistochemistry. Our experience in the treatment of UESL with epithelioid features suggests that complete surgical resection and postoperative auxiliary treatment may be beneficial for adult patients with similar conditions. As such, this case study has advanced our knowledge of UESL and the findings can be applied for the diagnosis and treatment of the disease in the future.

## Author contributions

**Conceptualization:** Peng Jiang.

**Funding acquisition:** Yahui Liu.

**Supervision:** Yan Jiao, Yahui Liu.

**Visualization:** Chunyuan Niu.

**Writing – original draft:** Peng Jiang.

**Writing – review & editing:** Peng Jiang.
